# Calibration of SO_2_ and NO_2_ Electrochemical Sensors via a Training and Testing Method in an Industrial Coastal Environment

**DOI:** 10.3390/s22197281

**Published:** 2022-09-26

**Authors:** Sofía Ahumada, Matias Tagle, Yeanice Vasquez, Rodrigo Donoso, Jenny Lindén, Fredrik Hallgren, Marta Segura, Pedro Oyola

**Affiliations:** 1 Airflux, Antonio Bellet 292, Santiago 7500000, Chile; 2 Independent Researcher, Antonio Bellet 292, Santiago 7500000, Chile; 3 IVL Swedish Environmental Research Institute, Aschebergsgatan 44, 41133 Gothenburg, Sweden; 4 Centro Mario Molina Chile, Antonio Bellet 292, Santiago 7500000, Chile

**Keywords:** calibration, electrochemical, relocation

## Abstract

Low-cost sensors can provide inaccurate data as temperature and humidity affect sensor accuracy. Therefore, calibration and data correction are essential to obtain reliable measurements. This article presents a training and testing method used to calibrate a sensor module assembled from SO_2_ and NO_2_ electrochemical sensors (Alphasense B4 and B43F) alongside air temperature (T) and humidity (RH) sensors. Field training and testing were conducted in the industrialized coastal area of Quintero Bay, Chile. The raw responses of the electrochemical (mV) and T-RH sensors were subjected to multiple linear regression (MLR) using three data segments, based on either voltage (SO_2_ sensor) or temperature (NO_2_). The resulting MLR equations were used to estimate the reference concentration. In the field test, calibration improved the performance of the sensors after adding T and RH in a linear model. The most robust models for NO_2_ were associated with data collected at T < 10 °C (R^2^ = 0.85), while SO_2_ robust models (R^2^ = 0.97) were associated with data segments containing higher voltages. Overall, this training and testing method reduced the bias due to T and HR in the evaluated sensors and could be replicated in similar environments to correct raw data from low-cost electrochemical sensors. A calibration method based on training and sensor testing after relocation is presented. The results show that the SO_2_ sensor performed better when modeled for different segments of voltage data, and the NO_2_ sensor model performed better when calibrated for different temperature data segments.

## 1. Introduction

The interest in citizen science focused on air quality has grown in parallel with the emergence of small, commercially available devices known as low-cost sensors. These sensors can also be adapted for the Internet of Things (IoT) and can thus be used in smart cities. Low-cost sensors have been suggested as promising tools to expand the spatial coverage of existing air monitoring networks and are also considered relevant to the paradigm shift of monitoring management, which is currently based on expensive instruments [1]. However, like any emerging technology, some limitations of low-cost sensors still need to be overcome. It has been recommended that the accuracy and quality of the data should be addressed when using low-cost sensors in citizen science projects [2,3].

Ubiquitous polluting gases, such as sulfur dioxide (SO_2_) and nitrogen dioxide (NO_2_), can be measured with low-cost sensors based on miniaturized electrochemical cells. SO_2_ and NO_2_ are irritating gases that affect people’s health and most countries regulate the public’s exposure to these pollutants through outdoor air quality standards [4]. The high coal consumption in homes and industry throughout the last century substantially increased the levels of SO_2_ in urban atmospheres. During the Great London Smog in 1952, one of the first air pollution disasters recorded by scientific instruments, the daily average concentrations of SO_2_ were as high as 0.7 ppm, equivalent to 1860 µg/m^3^ [5]. This higher-than-normal concentration was later identified to contribute to increased mortality rates after the event [6]. Although global SO_2_ emissions peaked in the 1990s and are progressively declining [7], NO_2_ emissions are rising globally due to the increasing number of fossil fuel vehicles [8]. Thus, both SO_2_ and NO_2_ still represent a pollution problem for many populations living in large cities or close to industrial settings.

Though the technology that enabled the development of electrochemical sensors emerged several decades ago [9], further refinement is required to enable low-cost sensors to achieve the same accuracy as reference air quality monitors [10]. Studies show that measurements made by low-cost electrochemical sensors are affected by temperature, humidity, and cross-reactions with other pollutants [11,12]. For example, ozone is a known interfering pollutant in NO_2_ electrochemical sensors. Thus, modern sensors include a scrubber or chemical filter that reacts with ozone molecules before the sample enters [13]. Natural sensor degradation is another feature that can lead to bias in sensor measurements. Long-term drift in sensor measurements is naturally caused by the aging and degradation of the inner components. At present, the average useful life of the current generation of low-cost electrochemical gas sensors does not exceed two years [14,15].

Existing guidelines on the use of low-cost sensors emphasize the importance of validating sensor performance through comparative measurements [16]. Most of the techniques explored in the literature are laboratory tests or field tests placing the sensors in parallel with reference instruments. Moreover, the post-processing of the raw data is recommended to obtain reliable measurements. Although there is no gold standard for data processing, a variety of statistical methods are used to estimate the reference measure; these approaches range from simple and multiple linear regression to machine learning algorithms such as random forests and neural networks; the latter have been shown to perform better than linear regression for sensor calibration [17,18].

Despite these advances in sensor calibration, statistical approaches can be sensitive to seasonal effects. For example, an algorithm based on data obtained in summer may not fit well for data obtained in another season [19]. Therefore, repeat calibrations during different seasons may be necessary for sensors used for a long time throughout the year. Moreover, although studies show that sensors from the same manufacturer often perform similarly, this cannot be guaranteed in all cases. Evidence indicates that a calibration model based on training data collected from an individual sensor may not work well when applied to another sensor, even a sensor of the same model and brand [19,20,21].

Consequently, data validation remains one of the most critical challenges in obtaining reliable measurements from any project involving low-cost sensors. Failure to calibrate a sensor or incorrectly assess sensor performance can lead to unusable data or incorrect analyses and conclusions. It has been postulated that data with poor quality or unknown quality are worse than no data, as they can lead to incorrect decisions [1]. However, the experiences published in the literature indicate that the data obtained from gaseous electrochemical sensors can be improved through calibrations that account for several variables, including relative humidity, temperature, and ozone.

Sensor calibration is being increasingly explored in citizen science as it allows the quality of the data to be validated. The following study presents a calibration method for low-cost electrochemical sensors subjected to training and test campaigns in the field. The calibration was based on multiple linear regression (MLR), which is a supervised machine learning method. Regression equations built for data separated into three segments allowed the low-cost sensors to estimate the reference measurements obtained with a scientific-grade instrument. The evaluated sensors were assembled on an IoT platform developed by the Swedish Luft-och-Vatten IoT initiative (LoV-IoT), which designed an air quality information system accessible to citizens. The training and test campaigns were developed in the industrial area of Quintero Bay, Chile, a territory known to be persistently polluted for many decades.

## 2. Materials and Methods

### 2.1. Principle of the Sensors

In electrochemical sensors, gas molecules diffuse through a porous membrane that is placed in contact with a metallic electrode, known as the working electrode (WE). This surface is where the analyzed gas is subjected to oxidation or reduction; i.e., gas molecules lose or acquire electrons at the WE. An equal and opposite reaction occurs at a second electrode, known as the counter electrode. For example, if oxidation occurs on the WE, reduction occurs on the counter electrode. The reduction of oxygen commonly occurs at the counter electrode in electrochemical sensors.

The electrodes are separated and immersed in an aqueous medium (electrolyte) that may include soluble salts, acids, or bases dissolved in a polar solvent such as water. The medium provides hydrogen ions (H^+^) that move through the aqueous solution. An acidic solution of 3–7 M sulfuric acid is one example of a sensor electrolyte.

**Figure 1:**
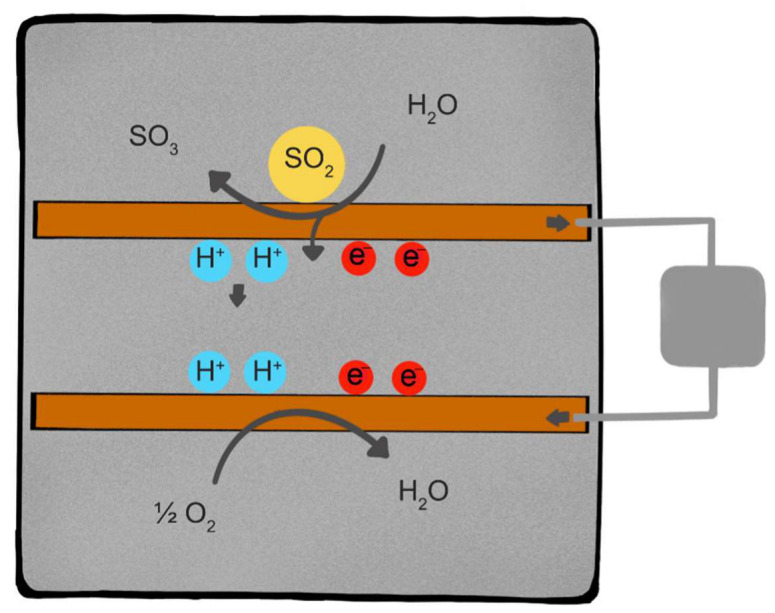
Working principle of a two-electrode electrochemical sensor for detection of SO_2._

The chemical reactions at the working electrode and counter electrode produce H^+^ and electrons (e^−^) that move to the opposite electrode. Electrons move through wires connected to the electrodes and an external circuit. The movement of electrons results in an electrical current that is detected by a pluggable chip, known as the ISB or individual sensor board. Figure 1 illustrates the working principle of a two-electrode cell used for sensing SO_2_.

Equations (1) and (2) are examples of the chemical reactions on which the electrochemical sensors used in this study are based:

SO_2_ + H_2_O ⇋ SO_3_ + 2 H^+^ + 2 e^−^ (1)

(1/2) O_2_ + 2 H^+^ + 2 e^−^ ⇋ H_2_O

NO_2_ + 2 H^+^ + 2 e^−^ ⇋ NO + H_2_O (2)

H_2_O ⇋ (1/2) O_2_ + 2 H^+^ + 2 e^−^

Most present-day electrochemical sensors also incorporate a third electrode, known as a reference electrode. This electrode is fixed to a constant potential by an external current produced in the ISB. An operational amplifier embedded in the ISB acts as a power source to send current into the electrochemical cell through the counter electrode, which has a floating potential. The current sent by the operational amplifier maintains the potential of the reference electrode at zero volts [22]. The current provided by the operational amplifier changes linearly to the current produced in the WE by the electrochemical reaction.

**Figure 2:**
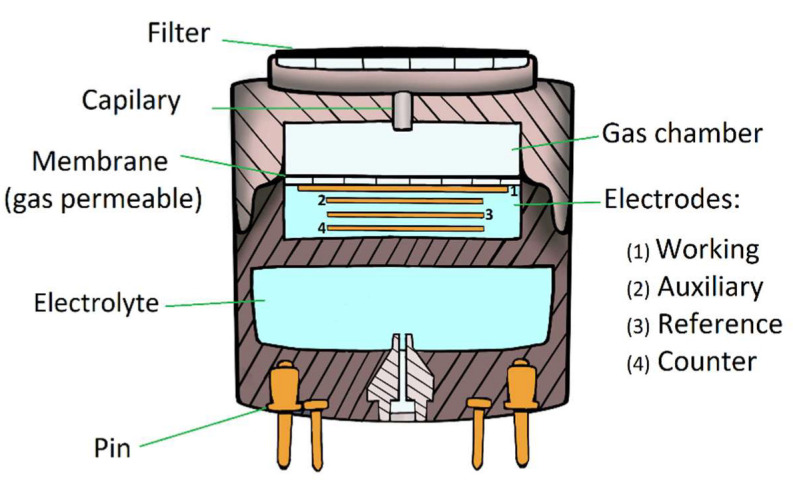
Components of a four-electrode electrochemical sensor for detection of air-polluting gases.

Some sensors also include an auxiliary electrode (AE) as a fourth electrode. The AE is used to correct the background current, also called zero current, which is produced when oxygen or impurities react in the electrolyte solution. The AE is isolated from the target gas, which allows the background currents to be determined. Consequently, the WE signal can be corrected by subtracting the zero current reported by the AE. Figure 2 shows a schematic diagram illustrating the principal components of a four-electrode electrochemical sensor [14].

### 2.2. Assembly

Two modules containing integrated sensors were assembled using the components listed in Table 1. The main parts are low-cost electrochemical sensors for SO_2_ and NO_2_ (Alphasense, Braintree, UK) with their respective individual sensor boards (ISB), an environmental sensor for T-RH, and an IoT microcontroller. The other components are a GPS module and two voltage transformers: 5 V for the electrochemical sensors and microcontroller, and 3.3 V for the T-RH sensor.

**Figure 3:**
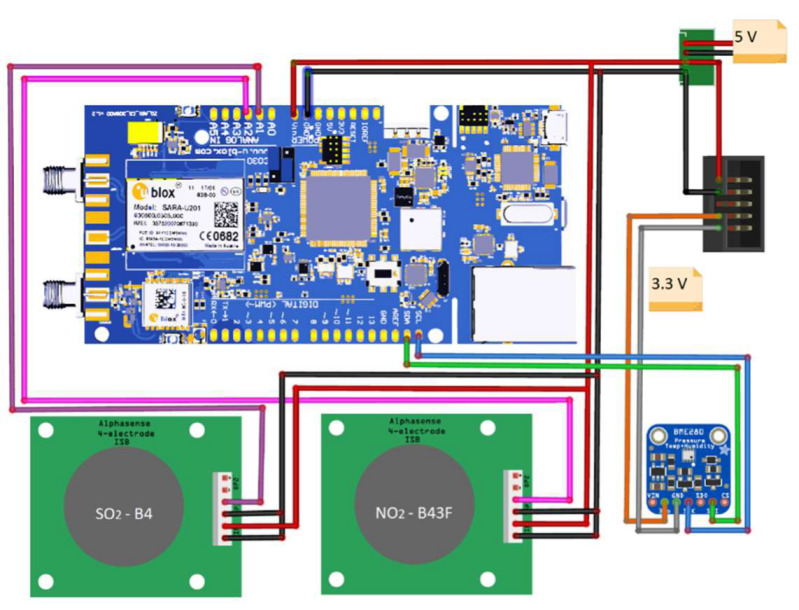
Module assembly and connections between the IoT controller board, sensors, and voltage regulators.

The individual parts were connected and assembled into an integrated module, as shown in Figure 3.

**Figure 4:**
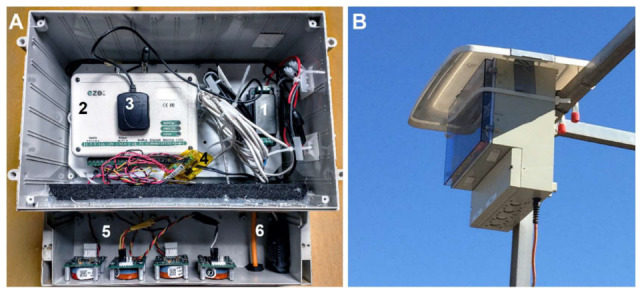
(**A**) Sensor module assembled inside the IP box. 1: Voltage transformer, 2: IoT microcontroller, 3: GPS, 4: T-RH sensor, 5: low-cost electrochemical sensors for SO_2_ and NO_2_, 6: power cable. (**B**) Sensor module deployed in the field.

An IP 65 plastic box (28 × 29 × 16 cm) was used to contain and protect the integrated module from water and dust. The SO_2_ and NO_2_ sensors were placed facing downwards at the bottom of the plastic case (Figure 4). The sensor units evaluated in this study had a date of manufacture of two months prior to use. The enclosure did not include any pumps or fans to generate air movement, so the sensing is considered to occur passively, i.e., by gases diffusing inside the electrochemical cell.

### 2.3. Data Transmission

The sensors generated an analog signal every second that was converted into a digital signal through an A/D converter added to the IoT microcontroller. The signals from both sensors were automatically averaged to one-minute values and aggregated into a JavaScript Object Notation (JSON). This file was sent to an Amazon Web Service (AWS) server, where the raw data were stored. The data in JSON were built with each sensor reading to show a timestamp and relevant data values. All timestamps were in Coordinated Universal Time (UTC) and each data value was self-describing with an ID, value, standard deviation, value type, and unit.

The sensor module was connected to the Internet through its 3G/4G mobile signal. The internet connection was used to send the sensor data to the AWS storage and to update the real-time clock using Network Time Protocol (NTP). The sensor communicated with the AWS using mutual transport layer security (TLS). The MQTT protocol was used to send and receive messages between the sensor and the server.

### 2.4. Field Deployment

**Figure 5:**
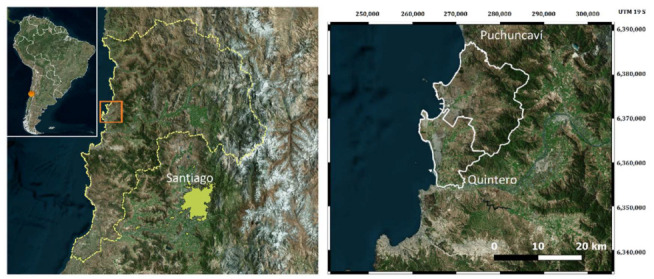
Location of the coastal cities where the sensor training and testing were conducted.

The sensor modules were subjected to field campaigns in two coastal cities, Quintero and Puchuncaví (Figure 5), in central Chile (lat. −32.84°, long. −71.47°). These cities are 110 km northwest of the country’s capital and together house a population of 56,000. A 2 km wide strip named the Ventanas Industrial Complex has been operating on the coastline for over half a century. This complex includes a copper smelter and a coal-fired power plant, both of which are characterized as significant sources of atmospheric SO_2_ and NO_2_.

The weather at the measurement site is associated with the Mediterranean coastal climate (Köppen-Geiger: Csb). In addition, the Pacific anticyclone influences the regional climate, enhancing several conditions associated with poor air quality, especially in colder months, when a low-altitude mixing layer and atmospheric stability become stronger. Both of these phenomena have been reported to significantly increase the concentrations of air pollutants in the area where the study was conducted [23].

At the social level, the communities report a long history of SO_2_ pollution episodes, as well as other environmental issues caused by industrial activity. This has led to these cities being termed a sacrifice zone. In this region, the impacts of industrial discharges and emissions on soil [24,25,26,27], sea water [28], and air quality [29] have been extensively evidenced.

**Figure 6:**
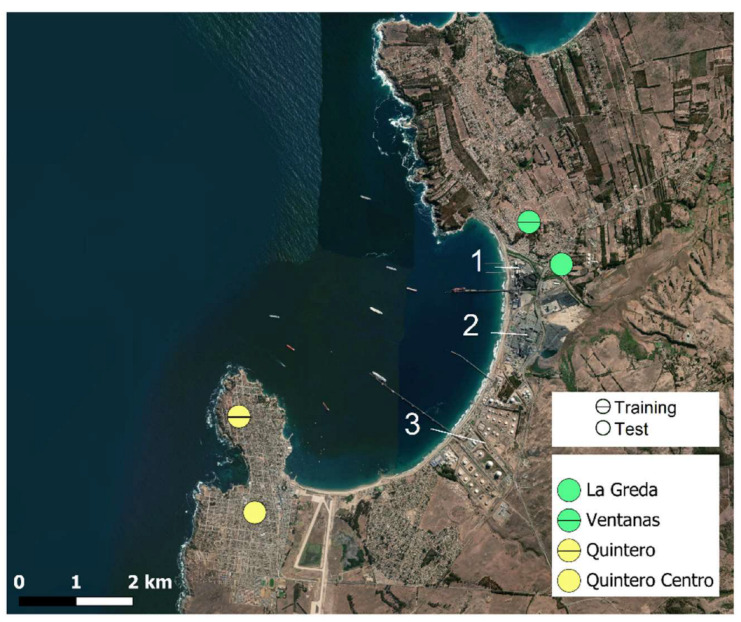
Location of the air quality monitoring stations used for training and testing the sensors. The major industrial facilities located along the bay are indicated by numbers: (1) coal-based power plant, (2) copper smelter-refinery, and (3) oil discharge and storage facility.

The sensor modules underwent training and testing in different field campaigns conducted between July and October 2019. Four regulatory monitoring stations were used for this purpose (Figure 6). At each station, the sensor module was fixed at roof level and located 2 m away from the inlet of the reference instruments for SO_2_ (Thermo Scientific 43i, Waltham, MA, USA) and NO_2_ (Thermo Scientific 42i). The station operator performed zero and span calibrations every three days on the reference equipment; the sensor modules only received sporadic cleaning and remote data revision. The details of the specific sensors used at each site are shown in Table 2.

### 2.5. Data Treatment

The output signals in millivolts (mV) from the working (WE) and auxiliary (AE) electrodes, as well as the data from the T and RH sensors, were visualized using Grafana and downloaded in csv format. The 1-min raw signal was averaged to 1-h values, and then the first three hours from connection to the electricity supply were discarded; this time frame is recommended for the stabilization of electrochemical sensors [10]. Measurements from the reference equipment were downloaded from the webpage that reports the information given by regulatory stations [30].

As the RH sensor integrated inside the module delivered flat data, we inferred that the RH sensor only measured the humidity conditions inside the plastic box. Thus, the RH data used for the calibration model were downloaded from a reference sensor installed at 10 m in a meteorological tower inside the industrial park (Met One 083E).

### 2.6. Calibration

Calibration was performed on the data obtained during the training period for the 1-h average values. The calibration method is based on multiple linear regression (MLR) equations developed from the downloaded output. The response variable in the MLR was the hourly average delivered by the reference monitor. To construct the MLR, the dataset was divided into a training set (70%) and a testing set (30%) based on the time series, with the first 70% of the time series used for training the model.

The MLR was performed independently for three groups of data segments, as defined by the voltage data for the SO_2_ sensor (Table 3) and by temperature for the NO_2_ sensor (Table 4). Each section relies on the quartiles of the data distribution. For example, data belonging to the first quartile were grouped in the first segment, data between the first and third quartiles were grouped in the middle segment, and data with values greater than or equal to the third quartile were grouped in the last (third) segment.

A Box–Cox transformation was performed in each data segment to normalize the distribution of the data; a normal distribution was obtained using an exponential known as the lambda power [31]. The lambda values used for normalization in this study are shown in Appendix A. After normalization, the studentized residuals were calculated via simple linear regression to identify potential outliers. A studentized residual is the value of a residual divided by the standard deviation of all residuals. A value of 3 was used as the threshold to identify outliers. The number of outliers for each data segment is shown in Table 3 and Table 4. Then, a separate MLR was applied to each data segment of the clean dataset. The regression formulas developed are shown in Equations (3) and (4):

SO_2_ reference (µg/m^3^) = β_0_ + β_1_ ×WE__SO2 sensor_ + β_2_ ×T + β_3_ ×RH + ε (3)

NO_2_ reference (µg/m^3^) = β_0_ + β_1_ ×WE__NO2 sensor_ + β_2_ ×T + β_3_ ×RH + ε (4)

where β_0_ is the intercept, β_1_, β_2_, and β_3_ are the estimated coefficients of the slopes in the explanatory variables, and ε is the error or residual of the regression.

The regression assumptions were evaluated by standard diagnostics, such as normality (Q-Q graphs), homoscedasticity (dispersion of standardized residuals), and the absence of multicollinearity (variance inflation factor). After diagnostic evaluation, the MLR was subjected to a 10-fold cross-validation by randomly splitting the testing data into 10 subsets to evaluate the MLR model in each subset. All of the analyses described above were conducted using R (version 3.6.2); the time series and scatterplots were constructed with IGOR Pro.

### 2.7. Calibration Performance

The sensor modules were relocated to a different monitoring station to assess the calibrated response (Figure 6). The relocation occurred two weeks after concluding the training campaign. The calibration performance was evaluated with standard metrics used to compare different models, for example, the R-squared coefficient of determination (R^2^) and metrics of calibration error, such as the mean absolute error (MAE) and root mean square error (RMSE); the formulas of these metrics can be found elsewhere [32].

R^2^ indicates linearity and the correlation between the model response and the measurements made with the reference instrument. R^2^ is usually preferred over Pearson’s correlation coefficient (r), which only describes the degree of correlation. In addition to correlation, R^2^ indicates the proportion of the response variable predicted by the explanatory variables. Both RMSE and MAE represent the difference between the value estimated by the calibration and the value reported by the reference instrument. These metrics are expressed in concentration units and provide information about the model’s tendency to overestimate or underestimate the reference value.

## 3. Results

### 3.1. SO_2_ Sensor Field Training and Calibration Performance

**Figure 7:**
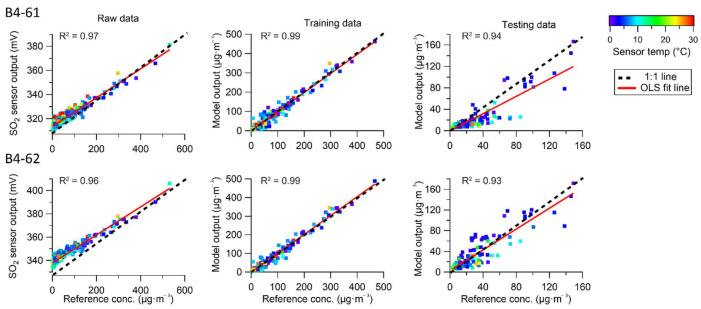
Scatterplots of SO_2_ (1-h average) measured in the training campaign by the reference instrument and the sensors (raw and calibrated response).

The SO_2_ sensors were only trained at the monitoring station “Quintero”, where the module with the sensors identified as B4-61 and B4-62 was placed. After the training campaign, the data from the sensors were compared with measurements delivered by the reference instrument. As shown in Figure 7, the raw response (mV) of the sensors correlated strongly with the reference measurement, confirming that the evaluated sensor produced data that change proportionally and linearly with the ambient SO_2_ concentration.

The degree of similarity between the data captured by different units of the same sensor model is termed reproducibility. During the training campaign, the two SO_2_ sensors presented robust reproducibility and low inter-unit variability, as the correlation between both units had an R^2^ of 0.99. However, the data series exhibited a difference of 25 mV between the values provided by each sensor unit. Raw data with a zero value were not identified in the data series; the minimum values recorded were 338 mV (sensor B4-61) and 313 mV (sensor B4-62).

**Figure 8:**
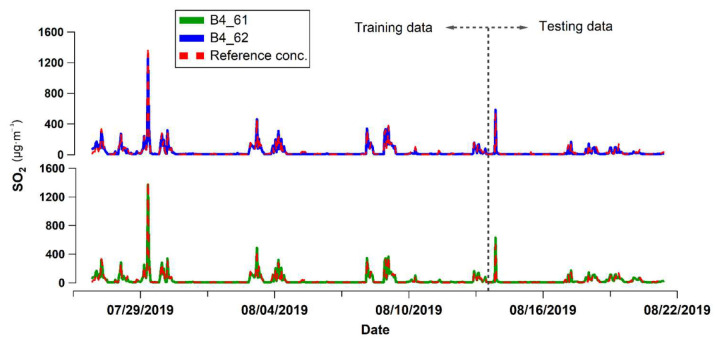
Time series of the 1-h average concentrations of SO_2_ measured in the training campaign by the reference instrument and the sensors (calibrated response).

The highest hourly concentration during the training period was recorded by the reference instrument at 1411 µg/m^3^ (19 July 2019), coinciding in time with the highest value in mV captured by the sensors. The sensors reported an hourly value of 483 and 458 mV (units B4-61 and B4-62, respectively) for this pollution event. Figure 8 illustrates the SO_2_ time series recorded by the reference instrument during the training field campaign; the maximum concentration recorded is visible.

As the maximum value influenced the linearity of the scatterplot, the SO_2_ peak is not included in Figure 7. Moreover, this maximum concentration was not used to construct the regression equation, as the model identified these data as an outlier. In addition, the scatterplots in Figure 7 compare the sensor data fitted using the calibration equation and their correlation with the reference instrument. A strong linear correlation is observed in the training dataset for both sensor units.

However, a weaker correlation between the sensor data and reference instrument was evident in the testing dataset, which exhibited a negative slope along the 1:1 line. This performance suggests that the calibration model generated for the SO_2_ sensor underestimate the reference concentration to some degree, particularly in scenarios of low concentrations recorded at low temperatures.

Multiple regression equations were built for the explanatory variables’ temperature, relative humidity and the sensor’s working electrode response in mV. The output is shown in Table 5, which presents the R^2^ and beta coefficients of the regressions obtained after applying each model to different data segments. The most robust regression was observed in the regression model for the data segments covering the highest voltages (R^2^ of 0.97). In contrast, the lowest R^2^ was observed in the lowest voltage segment.

R^2^ indicates the percentage of variance in the response variable that is explained by the set of predictor variables. Based on the performance in the training campaign, variables such as T, RH, and the raw response of the sensor (WE voltage) can predict the reference value to a large extent. However, these variables more strongly predicted the variance in data segments associated with higher voltages (regression models B and C).

Table 5 shows the beta coefficients of the regression, which represent the change in the response variable for each unit change in the predictor variable. The beta determined by the regression models was larger in the data segment associated with higher voltages, indicating that the calibration model is more sensitive at higher environmental concentrations of SO_2_. When the evaluated sensors captured an increase of 1 mV, the predicted or estimated referential concentration increased by 8 µg/m^3^. This behavior demonstrates that the data associated with higher voltages can influence a generalized calibration equation for low-cost electrochemical sensors. Therefore, construction of different regression equations for different data segments is appropriate.

Table 6 shows the significance level of the beta coefficient for each variable used to construct the regression model. The *p*-value was statistically significant (less than 0.1) for all three variables in the regression model, which indicates that ambient temperature, relative humidity, and the raw electrical signal significantly contribute to the estimation or prediction of the SO_2_ concentration provided by the reference instrument. However, the raw sensor response from the working electrode (WE) was the most significant variable in all of the regression models. This high significance is expected, since the electrical signal captured by the sensor is the most influential variable in the model, as evidenced by the higher beta coefficient of this variable compared to the other variables.

### 3.2. NO_2_ Sensor Field Training and Calibration Performance

**Figure 9:**
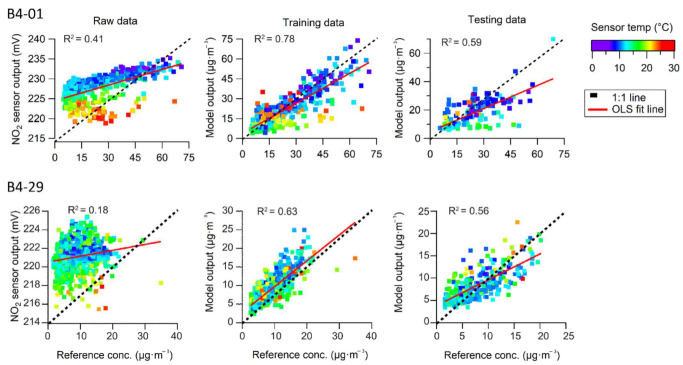
Scatterplots of NO_2_ (1-h average) measured in the training campaign by the reference instrument and the sensors (raw and calibrated response).

The low-cost NO_2_ sensors were trained at two monitoring stations. Sensor B4-01 was trained for 25 days at “Quintero” station, while sensor B4-29 was trained at “Ventanas” station for 45 days. The field measurements were taken in the winter months in the southern hemisphere. Overall, a weak correlation was observed between the raw data provided by the NO_2_ sensors and the reference instrument. The scatterplots in Figure 9 show the voltage data tended to cluster by temperature.

Three data clusters are visible above and below the 1:1 line for sensor B4-01. The dataset below the line was captured at higher temperatures (20 °C). The two data clusters above the line are associated with lower temperatures. After applying the calibration model, the sensor data correlated better with the reference instrument. The best correlation was observed in the training dataset, in which the data were well distributed and not grouped by temperature. A good fit was observed when validating the regression model in the testing dataset. However, the regression line is negative, indicating that the model is biased to underestimation.

**Figure 10:**
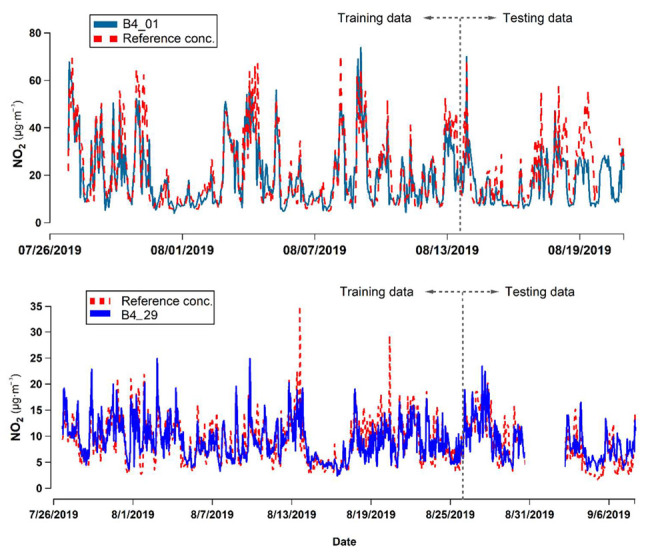
Time series of the 1-h average concentrations of NO_2_ measured in the training campaign by the reference instrument and the sensors (calibrated response).

No high-pollution events were recorded for NO_2_ during the training campaign. The hourly average concentrations were less than 70 µg/m^3^, which is expected in the urban environment of a medium-sized city. Figure 10 shows the time series of the data reported by the reference instrument and the sensors deployed in parallel. Although the timeseries are similar in shape and coincident with the cycles reported by the reference monitor, the calibration models applied to the NO_2_ sensors tend to underestimate the maximum concentrations.

The beta coefficients of the regressions performed for each data segment are shown in Table 7. The raw NO_2_ sensor data was segmented based on the temperature clusters observed. The regression models for both sensors resulted in the highest R^2^ values for the first data segment; thus, applying the calibration model resulted in the best linear fit for the lowest temperature range (9 or 11 °C), with an R^2^ above 0.80. In contrast, the linearity is lower and weaker in the data captured in the highest temperature data segment. Overall, these sensors tend to exhibit greater noise and fluctuations in the electrical signals generated at temperature conditions exceeding 20 °C.

The regression model for sensor B4-29 was built on the basis of four variables as the model with three variables resulted in a poor fit. The raw response of the auxiliary electrode (AE) was incorporated into the four-variable model. Background currents for NO_2_ sensors are captured by the AE. For sensor B4-29, the beta coefficient associated with the AE was higher in the low temperature range, and had a statistically significant *p*-value (Table 8). This suggests that including the data for the electrical signal of the AE may improve the performance of the calibration models for NO_2_ sensors for low-temperature conditions.

The significance levels for the beta coefficients of the regression models (Table 8) show that all of the variables in the models significantly contribute to the estimation of the reference NO_2_ concentration. The most influential variable in the models was the response from the WE, which had a significant *p*-value in all regression models (with the exception of model C for the B4-01 sensor). On the other hand, temperature and relative humidity were significant overall, with *p*-values greater than 0.1. However, both variables tended to have different *p*-values in different regression models and for different sensor units.

### 3.3. Calibration Performance at the Test Site

We evaluated the general performance of the calibration equations in a field test. For this test, the sensors were relocated to a monitoring station located within a radius of 1 km (Figure 6). The raw data obtained in this campaign were calibrated with the beta coefficients obtained for the MLR built in the training campaign, as well as Equations (3) and (4) presented in the Methods section.

Table 9 shows the performance metrics of the model applied to the data obtained in the field test. The root-mean-squared error (RMSE) was different for each sensor tested; the RMSEs were 12 and 16 µg/m^3^ for the two SO_2_ sensors, indicating medium-grade dissimilarity between the data produced by the calibrated sensor and the reference value. However, the R^2^ metric indicated a high correlation coefficient (R^2^ > 0.8) between the calibrated SO_2_ sensor and the reference data, suggesting that the MLR had a high capacity to capture the effects of ambient temperature and humidity on the sensor data. The R^2^ values suggested that the regression equations applied to the SO_2_ sensors explain more than 80% of the variability in the response estimated by the model, while the remaining 20% is the behavior attributed to other circumstances, such as electronic noise or interferences not accounted for in the model.

In other field calibrations, the Alphasense SO_2_ sensor (B4 series) was tested in Hawaii in areas close to volcanic activity. In that study, the sensors were calibrated through a hybrid model that included modeling SO_2_ concentrations below 50 ppb (133 µg/m^3^) using a mixed method that include K nearest neighbors and a linear regression model for data above 50 ppb [33]. That study reported similar metrics to ours, with an R^2^ of over 0.99, RMSE of 18–42 µg/m^3^, and MAE of 12–27 µg/m^3^.

For the NO_2_ sensors, although the metrics for the field test resulted in relatively low RMSE and MAE values, the R^2^ values were relatively weak and indicated low linearity with the reference instrument (R^2^ of 0.1–0.4). Analogous NO_2_ sensors have previously been reported to experience significant loss of sensitivity and signal deterioration over three months [34], which could explain the low linearity observed. Other studies using NO_2_ sensors of the B43F series reported mixed performance in the field (Table 10).

**Figure 11:**
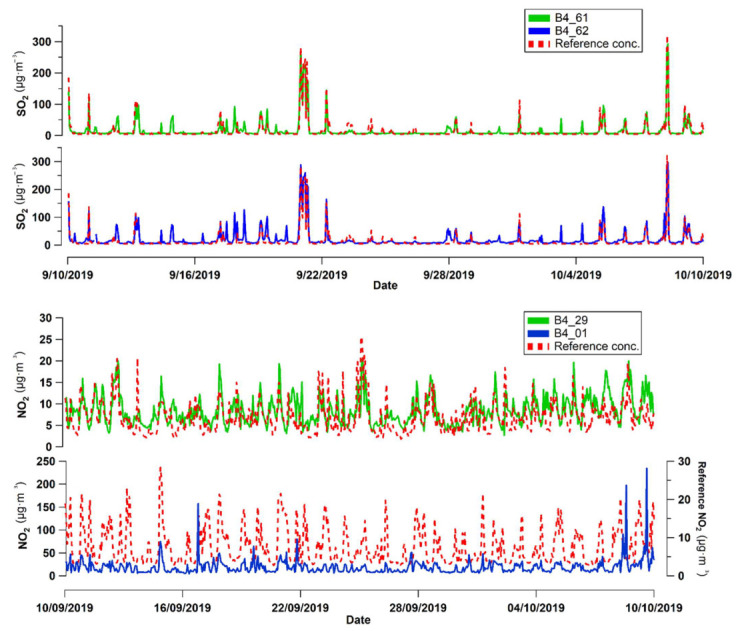
Time series of the 1-h average concentrations of SO_2_ and NO_2_ measured in the test campaign.

The time series recorded during the test campaign for the SO_2_ and NO_2_ sensors are shown in Figure 11. The SO_2_ sensors generated a series that matched the shape of the series reported by the reference instrument. However, some peak values captured by the sensors were not recorded by the reference equipment (e.g., data on 4 October). These peaks detected by the sensors and not the reference instrument cause the calibration to overestimate the average value (positive RMSE). The time series for NO_2_ were more dissimilar in shape to the reference instrument than the SO_2_ sensors. In particular, the poor performance of the B4-01 sensor is notable, as the time series significantly overestimates the reference value (e.g., peaks at the end of the series).

## 4. Discussion

Low-cost sensor technology is becoming widespread in citizen science, particularly for the analysis of air quality. This article presents a training and testing method to calibrate a custom-built air quality sensor assembled from electrochemical sensors for SO_2_ and NO_2_ (Alphasense B4 and B43F series). Training and testing were conducted in the field by placing the sensors next to the reference monitors. The raw responses from the electrochemical sensors (mV) were subjected to multiple linear regression (MLR) in three data segments. The data segmentation methodology and subsequent application of multiple regression allowed the construction of models that corrected the raw responses of the sensors. This method avoids poor data fitting, as the sensors tend to behave differently in different temperature or voltage ranges. For example, strong temperature dependence was evident for the NO_2_ sensors. The fit of the models for the data obtained at lower temperatures (i.e., the first quartile of data) generated higher R^2^. However, the fit of the NO_2_ sensor data and the reference measurements became weaker as the temperature increased. Like humidity in the air, temperature can affect sensor performance. It has been shown that both exert changes in the electrode–electrolyte interface [12]. In addition, the weaker performance may be due to the effect of temperature on the gas in the electrolyte liquid. At lower temperatures, the gas is expected to be more soluble in the electrolyte, thus enabling a better estimate of ambient NO_2_ concentrations.

The tests showed that the response of the sensors is dependent on temperature. Therefore, MLR regression models that include this variable may result in better calibration. However, the calibration does not perform well in conditions of T above 30 °C, as the responses of the sensors lose linearity. In our study, the cross-reaction with O_3_ was assumed to not affect the calibration method, as long as the NO_2_ sensor has a built-in chemical filter that absorbs ozone (0.5 ppm O_3_/h in B43F series). However, the sensitivity of the low-cost electrochemical sensors evaluated in this study could change over the course of the field tests due to natural degradation.

The sensitivity of the sensors is expected to decrease over time due to the natural degradation of the electrodes. In addition, sensitivity losses can be caused by evaporation of the electrolyte liquid. This natural degradation translates into a drift in the baseline measurements made by the sensors; such drift can occur within four months of monitoring. The manufacturer of the sensors used in this study indicates a 50% decay in sensitivity after two years, but other studies suggest that there is a need for recalibration after the first four months [15]. In this case, where the field tests were extended over 4 months, a loss of sensitivity due to sensor degradation is largely not expected.

The aqueous solution in the electrochemical cell loses water in low humidity conditions and absorbs water in high humidity conditions. The sensors work normally at humidity values between 15% and 90%; outside this range, performance is affected by dryness or by excess water, with the latter causing the electrolyte to overflow outside the sensor. The sensor continuously stabilizes at its local humidity, with the ideal RH condition of 60%. Temperature also affects water loss, since the water in the electrolyte evaporates faster at higher temperatures. In this study, the environmental conditions of the coastal area where the sensors were located exhibited temperature and humidity ranges within the optimal operating ranges of the sensors; therefore, electrolyte evaporation is not expected to have occurred.

In agreement with the findings of our study, temperature and relative humidity are described as significant variables for the calibration of electrochemical sensors [11,17]. However, the performance of a calibration model is related to the statistical technique used. In our study, MLR resulted in good performance; nevertheless, other regression models, though in the form of parametric and quadratic regressions, have also resulted in calibration that improved sensor performance in terms of the raw response [41,42].

Overall, this study demonstrates that multiple linear regression can be used to calibrate low-cost electrochemical sensors. The performance of calibration is better when testing is conducted in a limited geographical area [33]. One limitation of this study is that we did not experimentally verify any loss of sensitivity of the sensors due to electrolyte degradation. One simple approach would be to weigh the sensor before and after it is deposited in the field.

The evaporation of the electrolyte has been proven to contribute to loss of the area of interfacial contact with the electrode surface [12]. In the event that the sensors are used for a long period of time, our method faces the limitation of not incorporating the effect of degradation-related drift in the sensor data into the model. Drift in measurements over longer periods represents a factor that decreases the performance of a calibration model. Thus, drift should be analyzed in other monitoring projects that aim to collect data using low-cost sensors.

## 5. Conclusions

This study presented a calibration model based on training field evaluations and sensor testing after relocation. Multiple linear regression models were built to estimate the concentrations measured by the reference instrument.

Building a regression model for different data segments allowed the responses of the sensors to be fitted better. The correlations with the reference instrument were robust (NO_2_: R^2^ > 0.6; SO_2_: R^2^ > 0.97). The performance of the SO_2_ sensor was better when modeled for different segments of voltage data, while the NO_2_ sensor model performed better when calibrated for different temperature data segments.

The SO_2_ sensor detected a high-pollution event, demonstrating the potential of low-cost sensors as an early warning system. Thus, low-cost sensors represent promising tools for industrial environments where rapid changes in pollutant concentrations may occur.

## Figures and Tables

**Table 1:** Main components of the integrated air quality sensor.

Component	Module 1	Module 2
NO_2_ sensor	Alphasense NO2B43F	Alphasense NO2–B43F
SO_2_ sensor	Alphasense SO2–B4	-
T and RH sensor	Transistor L7805CV	Bosh BME 280 Adafruit
IoT microcontroller	ezeio, eze Systems	u-blox C030 U201 + Arduino

**Table 2:** Identification of the sensors deployed in the monitoring stations.

	Module 1	Module 2
Sensor ID	SO2_B4-61 SO2_B4-62 NO2_B4-01	NO2_B4-29
Training station	Quintero	Ventanas
Testing station	Quintero Centro	La Greda

**Table 3:** Dataset from the training campaign disaggregated by voltage range.

SO_2_	Data	Voltage	Data	Outliers
Sensor	Group	(mV)	n	n
B4-61	A	<338	165	32
	B	338 to 346	173	29
	C	>346	90	5
B4-62	A	≤313.5	142	15
	B	331.5–321.5	196	25
	C	≥321	92	6

**Table 4:** Dataset from the training campaign disaggregated by temperature range.

NO_2_	Data	Temperature	Data	Outliers
Sensor	Group	(°C)	n	n
B4-01	A	≤11	133	6
	B	11–16.5	180	18
	C	≥16.5	117	23
B4-29	A	≤9	233	18
	B	9–14	294	19
	C	≥14	227	27

**Table 5:** Regression coefficients of the MLR applied to different segments of data (voltage) generated by the SO_2_ sensors.

SO_2_	Regression	Voltage	R^2^	Intercept	β_1_ WE	β_2_ T	β_3_ RH
Sensor	Model	mV		µg∙m^−3^	µg∙m^−3^/mV	µg∙m^−3^/°C	µg∙m^−3^/%
B4-61	A	≤338	0.38	3.07	−0.008	−0.002	−0.001
	B	338–346	0.79	11.8	−0.032	−0.001	−0.0003
	C	≥346	0.97	−3020	8.93	−1.89	0.15
B4-62	A	≤313.5	0.18	4.29	−0.012	−0.00004	−0.0009
	B	313.5–321.5	0.72	10.2	−0.032	−0.00007	−0.004
	C	≥321	0.97	−2745	8.86	−3.14	−0.19

**Table 6:** Significance levels of the *p*-values for the variables used in the regression models for the SO_2_ sensors (*** = 0.001, * = 0.1).

SO_2_ Sensor	Regression Model	Intercept	WE	T	RH
B4-61	A	***	***	***	***
	B	***	***	***	*
	C	***	***	***	*
B4-62	A	***	***	*	***
	B	***	***	*	***
	C	***	***	***	*

**Table 7:** Regression coefficients of the MLR applied to different segments of data (temperature) generated by the NO_2_ sensors.

NO_2_	Regression	Temp	R^2^	Intercept	β_1_ WE	β_2_ T	β_3_ RH	β_4_ AE
Sensor	Model	(°C)		µg∙m^−3^	µg∙m^−3^/mV	µg∙m^−3^/°C	µg∙m^−3^/%	µg∙m^−3^/mV
B4-01	A	≤11	0.88	−162	0.73	0.078	−0.017	
	B	11–16.5	0.76	6.74	−0.026	−0.003	0.00003	
	C	≥16.5	0.59	1.07	−0.0008	−0.026	−0.0012	
B4-29	A	≤9	0.85	23.3	2.1	0.07	0.019	−2.17
	B	9–14	0.78	11.7	0.075	−0.0004	−0.004	−0.013
	C	≥14	0.61	17.2	0.065	0.075	−0.005	0.017

**Table 8:** Significance levels of the *p*-values for the variables used in the regression models for the NO_2_ sensors (*** = 0.001, ** = 0.05, * = 0.1).

NO_2_ Sensor	Regression Model	Intercept	WE	T	RH	AE
B4-01	A	***	***	**	**	
	B	***	***	*	*	
	C	*	*	***	**	
B4-29	A	***	***	*	*	***
	B	***	***	*	***	**
	C	***	***	***	***	**

**Table 9:** Performance metrics of the calibrated sensors in the testing period. RMSE and MAE are expressed in concentration units (µg m^3^).

	Sensor	R^2^	RMSE	MAE
SO_2_	B4-61	0.85	12.35	6.29
	B4-62	0.81	16.52	8.90
NO_2_	B4-01	0.18	18.48	11.40
	B4-29	0.45	3.84	2.99

**Table 10:** Results from other studies evaluating NO_2_ sensors (B43F series) calibrated with reference instrumentation.

Reference	Location	RMSE (µg/m^3^)
[35]	Dublin (Ireland), Bologna (Italy)	0.19–0.23
[36]	Arizona (U.S.)	11.3–18.8
[37]	Metz (France)	4.7–6.3
[38]	Guangzhou (China)	28.3–53.4
[39]	Parma (Italy)	5.6–14.1
[40]	Seattle (U.S.)	3

## Data Availability

Data supporting the reported results will be provided upon readers’ request.

## Appendix A

**Table A1:** Lambda values used in data normalization by Box–Cox transformation.

SO_2_	Regression	λ
Sensor	Model	
B4-61	A	−0.515
	B	−0.252
	C	1
B4-62	A	−0.535
	B	−0.6
	C	1
**NO** **_2_**	**Regression**	**λ**
**Sensor**	**Model**	
B4-01	A	0.515
	B	−0.131
	C	−0.515
B4-29	A	0.838
	B	0.191
	C	0.252
